# Murine Melanoma-Infiltrating Dendritic Cells Are Defective in Antigen Presenting Function Regardless of the Presence of CD4^+^CD25^+^ Regulatory T Cells

**DOI:** 10.1371/journal.pone.0017515

**Published:** 2011-03-03

**Authors:** Haley Ataera, Evelyn Hyde, Kylie M. Price, Patrizia Stoitzner, Franca Ronchese

**Affiliations:** Malaghan Institute of Medical Research, Wellington, New Zealand; Universität Würzburg, Germany

## Abstract

Tumor-infiltrating dendritic cells are often ineffective at presenting tumor-derived antigen *in vivo*, a defect usually ascribed to the suppressive tumor environment. We investigated the effects of depleting CD4^+^CD25^+^ “natural” regulatory T cells (Treg) on the frequency, phenotype and function of total dendritic cell populations in B16.OVA tumors and in tumor-draining lymph nodes. Intraperitoneal injection of the anti-CD25 monoclonal antibody PC61 reduced Treg frequency in blood and tumors, but did not affect the frequency of tumor-infiltrating dendritic cells, or their expression of CD40, CD86 and MHCII. Tumor-infiltrating dendritic cells from PC61-treated or untreated mice induced the proliferation of allogeneic T cells *in vitro*, but could not induce proliferation of OVA-specific OTI and OTII T cells unless specific peptide antigen was added in culture. Some proliferation of naïve, OVA-specific OTI T cells, but not OTII T cells, was observed in the tumor-draining LN of mice carrying B16.OVA tumors, however, this was not improved by PC61 treatment. Experiments using RAG1^−/−^ hosts adoptively transferred with OTI and CD25-depleted OTII cells also failed to show improved OTI and OTII T cell proliferation *in vivo* compared to C57BL/6 hosts. We conclude that the defective presentation of B16.OVA tumor antigen by tumor-infiltrating dendritic cells and in the tumor-draining lymph node is not due to the presence of “natural” CD4^+^CD25^+^ Treg.

## Introduction

The presence of dendritic cells (DC) in numerous human [1] and murine tumors [2], [3] is well established. The role of these tumor-infiltrating DC (TIDC) in the tumor-specific immune response, and their value as indicators of disease progression, are, however, unclear [4], [5]. A number of studies have shown that TIDC have poor tumor antigen presenting function *in vitro* and *in vivo* [3], [6]–[13]. Tumors represent an immunosuppressive environment containing a range of inhibitory mechanisms such as decreased inflammatory cytokines, increased anti-inflammatory cytokines [14], [15] and increased Treg infiltration [16], [17], which are likely to affect the function of local T cells [18] as well as DC [7].

The term “regulatory T cells” (Treg) refers to a range of cells which express distinct phenotypes but share a common suppressive function [19]. Among these suppressive populations, CD4^+^CD25^+^ “natural” Treg (from now on referred to as “Treg”) are prominent due to their essential role in the maintenance of self-tolerance. Treg require expression of the transcription factor Foxp3 for their normal development in the thymus, and are thought to require antigen specific activation by DC in order to acquire effector function in the periphery [20], [21]. Mice in which Treg function is defective develop severe autoimmunity that can be prevented by the transfer of CD4^+^CD25^+^ T cells [22], [23]. Treg suppress T cell proliferation and degranulation, inhibit CTL function, and may cause T cell death through production of the anti-inflammatory molecules adenosine, transforming growth factor (TGF)-β and interleukin (IL)-10, and inhibition of IL-2 transcription in T cells (reviewed in [21]). It has further been proposed that Treg may cause cytokine deprivation-induced apoptosis of target T cells, or even directly kill target cells using granzyme B and perforin [21], [24], [25]. Treg have been shown to inhibit production of inflammatory cytokines such as Interferon-γ [26], Tumor Necrosis Factor-α [27] and the cytolytic granule proteins perforin and granzymes [28].

In addition to their suppressive effect on T cells, Treg may also suppress macrophages, natural killer (NK) cells, B cells [21] as well as DC. Studies in non-obese diabetic mice have shown that Treg can inhibit the expression of the DC activation markers CD40, CD80, CD86 and MHCII, both *in vitro* and *in vivo* [21], [29], [30], and interact directly with DC during immune responses [31], decreasing the interaction time between effector T cells and DC [32]. A number of studies have also shown that Treg indirectly control DC homeostasis *in vivo* [23], [33], [34]. In tumor bearing mice, Treg have been shown to induce DC death in the lymph node (LN) [35], but little information is available on whether Treg may affect the number, phenotype or function of DC within the tumor context. Effects on DC antigen uptake and/or function might result in diminished T cell activation and effector differentiation within the tumor [36]. Effects on DC migration and/or function might also lead to decreased antigen presentation in the draining LN. In this paper we use a B16.OVA melanoma model to investigate and report the effects of Treg depletion on the antigen presenting function of TIDC *in vitro* and *in vivo*.

## Materials and Methods

### Ethics statement

All experimental procedures were approved by the Victoria University Animal Ethics Committee (permits 2004R6M and 2007R4M) and carried out according to Institutional guidelines.

### Mice

All mice were bred at the Malaghan Institute of Medical Research Biomedical Research Unit. C57BL/6J mice were originally from Jackson Laboratories, Bar Harbor, ME, while CD45-congenic B6.SJL-Ptprc^a^Pep3^b^/BoyJArc (CD45.1) were from the Animal Resource Centre, Canning Vale, Western Australia. OTI and OTII mice expressing transgenic T cell receptors (TCR) specific for K^b^+OVA_257–264_ and I-A^b^+OVA_323–339_, respectively, were obtained with the permission of F. Carbone, Melbourne University, Australia. Foxp3GFP mice [37] were obtained from Prof. A. Rudensky, University of Washington, USA; hemizygous males were used in all experiments. B6.129-Rag1^tm1^Mom mice (RAG1^−/−^) were from the Walter and Eliza Hall Institute, Melbourne, Australia. Mice were used when 6–8 weeks old and gender-matched within experiments.

### Media and reagents

Cells were cultured in Iscove's Modified Dulbecco's Medium (IMDM) supplemented with 5% Fetal Bovine Serum (FCS), 100 U/ml penicillin, 100 µg/ml streptomycin and 50 µM 2-mercapto-ethanol (all from Invitrogen, Auckland, NZ). Synthetic peptides were from Mimotopes Pty Ltd (Clayton, Victoria, Australia).

### Antibodies and flow cytometry

Monoclonal antibodies specific for murine CD11c, MHCII, F4/80, CD25 and CD3 were affinity-purified from hybridoma culture supernatants using protein G-Sepharose (Pharmacia Biotech, Uppsala, Sweden) and were left purified or conjugated to various fluorophores. Fluorescent antibodies specific for CD45, CD8α CD11b, CD11c, MHCII, CD86, CD40 and CD25 were from BD-Pharmingen (San Diego, USA). Anti-CD45 and anti-Foxp3 intracellular staining kits were from eBioscience (San Diego, USA). All reagents were used according to manufacturer's instructions. Live cells were identified by FSC and SSC properties.

### Treg depletion

Mice were given two intraperitoneal (i.p.) injections of 100 µg purified PC61, 3 days apart [38]. Where applicable, tumor inoculation was carried out one day following the last PC61 injection. Treg depletion was estimated by flow cytometry of tail blood samples.

### Isolation of T cells

LN from OTI or OTII mice were pressed through a 70 µm cell strainer (BD Falcon, USA). CD8^+^ T cells were positively selected from OTI cell suspensions using anti-CD8α-MACS beads (Miltenyi Biotec, Germany) and magnetic sorting. Effector CD4^+^ T cells were first depleted of CD25^+^ cells by incubation with anti-CD25-PE followed by anti-PE MACS beads and negative magnetic selection, followed by positive selection with anti-CD4-MACS beads. OTI and OTII cell populations were routinely >95% and ∼80% pure, respectively.

### Tumor experiments

The B16.OVA cell line [39] was kindly provided by Drs. Roslyn Kemp and Dick Dutton, Trudeau Institute, USA, while B16.F1 was from ATCC, Manassas, VA, USA. Mice were injected subcutaneously (s.c.) with 10^5^ tumor cells into the flank, and tumor growth was measured every 2 days using calipers. Tumors were removed, weighed, dissociated using tweezers and digested with 0.4 mg/ml Liberase CI (Roche Applied Science, Mannheim, Germany) and 0.1 mg/ml DNase I (Roche) for 30 min at 37°C. Digestion was stopped with 10 mM EDTA for 5 min at 37°C and suspensions pressed through 70 µm cell strainers.

### Isolation and quantification of TIDC

Tumor cell suspensions were prepared, counted using trypan blue, and the frequency of live cells that were CD45^+^, CD11c^hi^ was determined by flow cytometry. For sorting experiments, leukocytes were enriched to ∼20% using anti-CD45-PE, anti-PE MACS beads, and magnetic selection. Cells were then incubated with anti-CD11c and electronically sorted to obtain a population that was >96% pure.

### *In vitro* suppression assay

Tumor cell suspensions from Foxp3GFP mice were enriched for CD4^+^ cells using anti-CD4-MACS beads and magnetic selection. Cells were then incubated with anti-CD45-PE, and GFP^+^CD45^+^ cells were electronically sorted to approximately 98% CD45^+^GFP^+^. These Treg were cultured at differing ratios with a constant number of DC (2.4×10^3^/well), CD4^+^ CD25^−^ effector T cells (4×10^4^/well), and 1 µg/ml anti-CD3 for 3 days. ^3^H-thymidine (1 mCi/ml, Amersham, Aylesbury, UK) was added during the last 6 h of culture before harvesting on a Tomtec cell harvester (Orange, CT, USA) and counting on a Betacounter (Wallac, Turku, Finland) to determine the amount of proliferation.

### *In vitro* proliferation assays

TIDC were sorted and titrated in duplicate into 96 well U bottom plates containing 2×10^5^ purified OTI or OTII T cells in a total volume of 200 µL. After 3 days, 1 µCi ^3^H-thymidine was added to each well for 6 hours. Cells were harvested and counted as above.

### Carboxyfluorescein succinimidyl ester (CFSE) labeling

Single cell suspensions (5×10^6^ cells/ml) were incubated for 10 min at 37°C with 0.2 mM CFSE (Molecular Probes, Eugene, Oregon). The reaction was stopped by adding one volume of FBS. Cells were washed once with complete media and twice with PBS.

### *In vivo* proliferation assays

B6.SJL mice were inoculated with tumor and 13 days later were injected s.c. in the forearm with 2×10^5^ DC that were loaded with 1 uM OVA_257–264_ (SIINFEKL) or left untreated. One day later, mice were injected i.v. with 1.5×10^6^ OTI and 1.5×10^6^ OTII T cells labeled with CFSE. Tumor-draining and non-draining LN were removed 3 days after T cell transfer, and analyzed for T cell proliferation by flow cytometry.

## Results

### Tumor-derived CD25^+^ Foxp3^+^ Treg suppress T cell proliferation *in vitro* and are depleted by PC61 treatment

**Figure 1:**
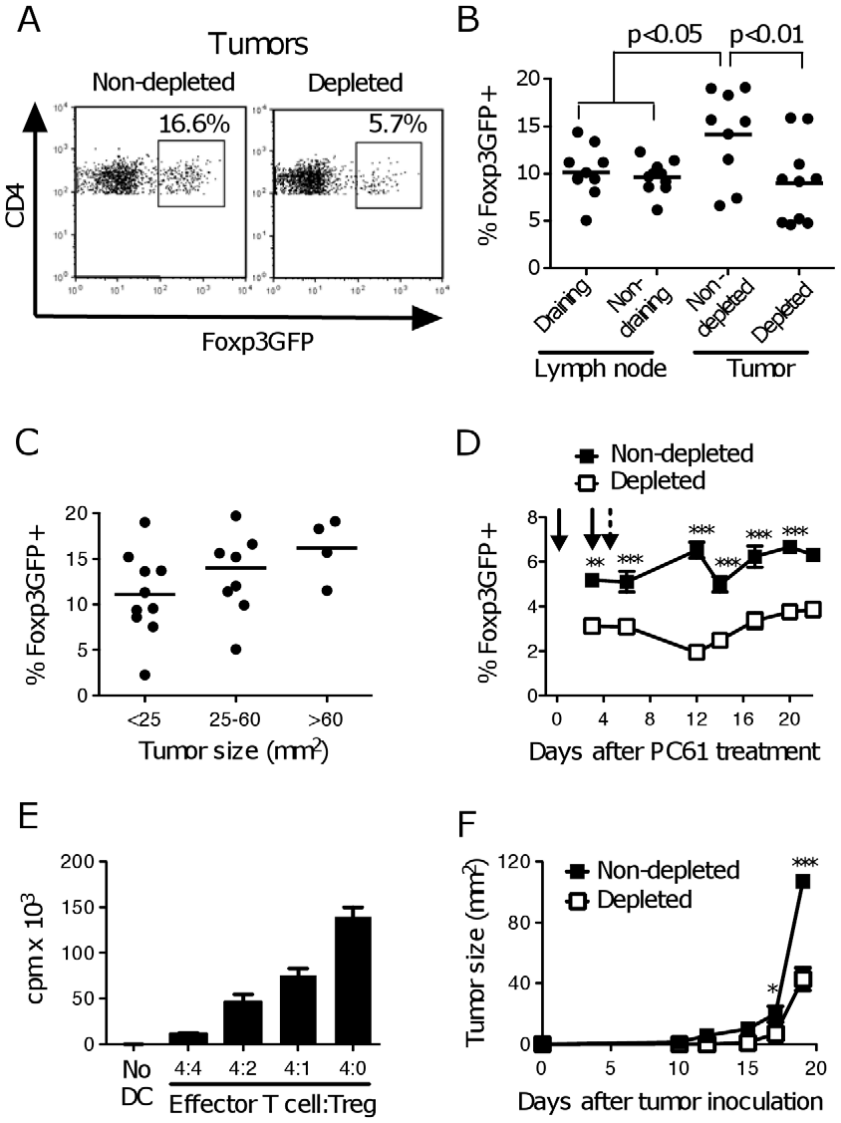
Tumor-infiltrating Foxp3^+^ Treg are suppressive *in vitro* and affect tumor growth. Foxp3GFP mice were treated with PC61 or left untreated, and injected with B16.OVA tumors s.c. Tissues were removed for analysis at different times after tumor challenge. (**A**) The frequencies of Treg in tumors from non-depleted mice, or mice depleted of Treg by PC61 treatment, were determined by flow cytometry. Each panel refers to an individual representative mouse. (**B, C**) Frequencies of Foxp3GFP^+^ cells in different tissues (B) and in tumors of different sizes (C), determined as shown in panel A. Each dot represents one mouse. Data are from 2 experiments, each with 4–5 mice per group, collected 14–17 days after tumor inoculation. Average values are shown by a horizontal line, *p* values were calculated using one-way ANOVA. (**D**) Mice were treated with PC61 (solid arrows) or left untreated, and injected with B16.OVA (arrow with broken line). Mice were bled over time to monitor the frequency of Foxp3GFP^+^ Treg within the peripheral CD4^+^ population. Average ± SEM for groups of 5 mice are shown. (**E**) CD4^+^ Foxp3GFP^+^ Treg were sorted from tumors and titrated into wells containing constant numbers of purified CD4^+^CD25^−^ effector T cells, DC, and anti-CD3. Proliferation was measured 3 days later. Bars represent average ± range for duplicate samples. (**F**) C57BL/6 mice were treated with PC61 as in D or left untreated, and inoculated with tumor. Average tumor sizes ± SEM are shown. Results are from one of 4 repeat experiments that gave similar results. Values of *p* (where *<0.05 and ***<0.001) were calculated using a non-parametric one-way ANOVA with a Dunn's post-test.

To establish whether Treg were present in B16.OVA tumors, we used Foxp3GFP mice where natural Treg can be easily identified by Green Fluorescent Protein (GFP) expression (Figure S1 and [37]). In B16.OVA tumors, ∼14% of the CD4^+^ T cell population was Foxp3GFP^+^, as opposed to the ∼10% observed in the tumor-draining and non-draining LN (Figure 1A and 1B), and their frequency increased during tumor growth (Figure 1C). This Foxp3GFP^+^ population may also include “induced” Treg, but is unlikely to include Tr1 cells which are Foxp3GFP^−^ and CD25^−^ [19].

In tumor-bearing mice, PC61 treatment routinely achieved a 40–70% depletion of the Foxp3GFP^+^ population in blood, which was maintained throughout the experiment (Figure S1A and Figure 1D). The remaining Foxp3GFP^+^ population was essentially CD25^−^ until the very final stages of tumor growth (Figure S1B). The intratumoral Foxp3GFP^+^ population was still reduced in PC61-treated mice compared to untreated mice at day 17 (Figure 1A and 1B). As also shown by others [21], [40], tumor derived Foxp3GFP^+^ cells were able to suppress the proliferation of conventional T cells *in vitro* (Figure 1E). Treatment with PC61 *in vivo* resulted in a significant delay in tumor growth, and by day 20 tumors in Treg-depleted mice were only about one third of the size in the non-depleted group (Figure 1F). Together, these data suggest that Treg are present in B16.OVA tumors from an early stage, and may contribute to their rapid growth.

### Treg do not affect the frequency or phenotype of DC in B16.OVA tumors and in draining LN

**Figure 2:**
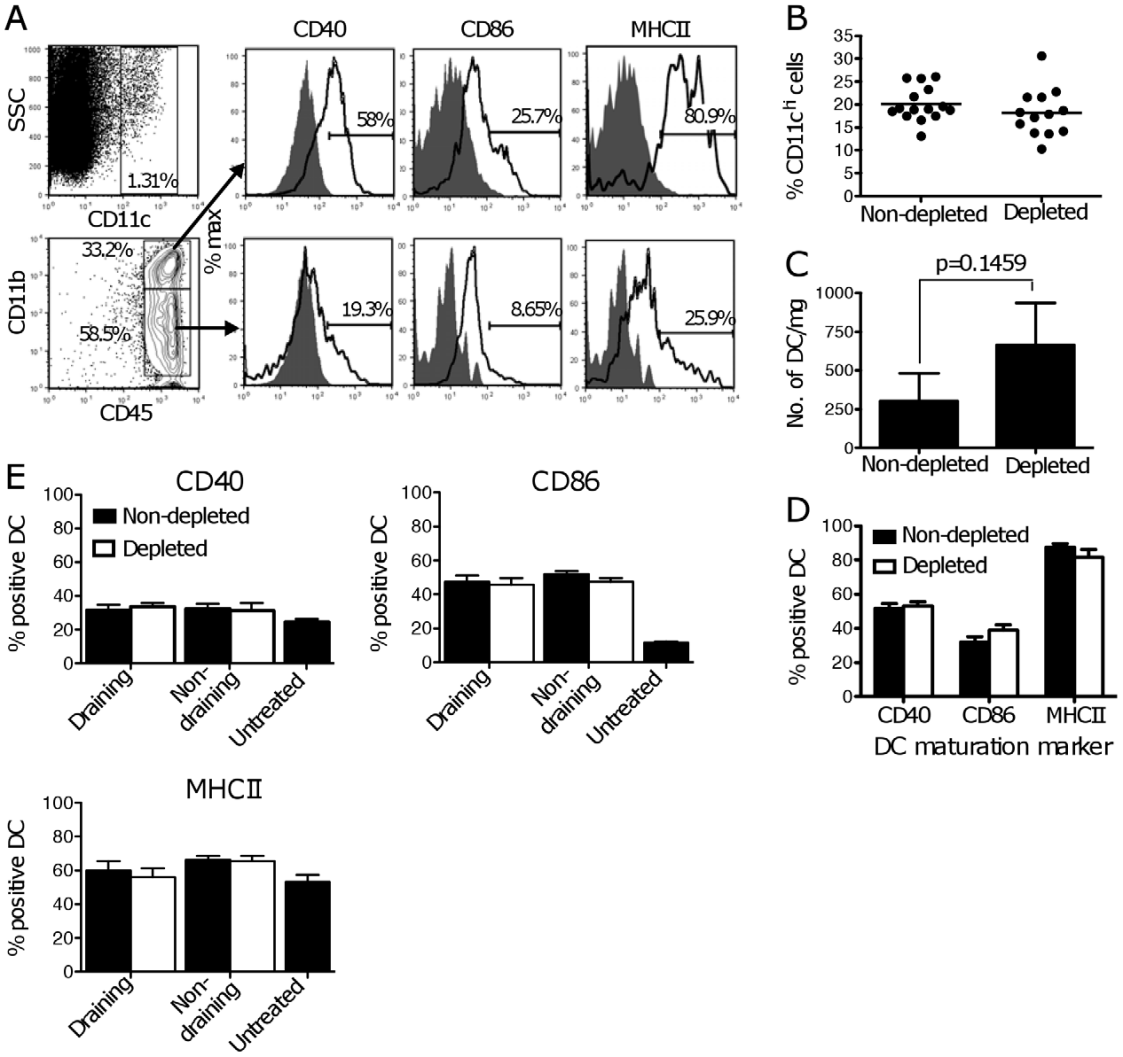
Treg depletion does not affect the frequency or phenotype of TIDC and DC in LN. C57BL/6 mice were treated with PC61 or left untreated, and injected with B16.OVA s.c. Tumors and LN were removed for analysis at different times after tumor challenge. (**A**) Gating strategy used to identify TIDC (CD45^+^, CD11c^hi^, CD11b^hi/int^) and assess expression of CD40, CD86 and MHCII. Fluorescence-minus-one controls (CD40) and isotype controls (CD86 and MHCII) are shown as grey filled histograms, while empty histograms show marker expression. Percentages of cells expressing the relevant markers are shown. (**B**) Frequencies of DC in tumors, expressed as % CD11c^hi^ cells in the CD45^+^ population. Each dot corresponds to one mouse. Data are from 3 experiments, each with 4–5 mice per group. (**C**) Numbers of DC per mg of tumor when tumors in both groups are of similar size (200–300 mg). Bars show the average number of DC+SE from 2 experiments each with 3–4 mice per group. The average tumor size in the two groups was similar. (**D**) Percentages of TIDC expressing the indicated maturation markers in Treg-depleted and non-depleted mice. Bars show the average+SEM for a compilation of 3 independent experiments each with 5 mice per group. (**E**) Expression of maturation markers on DC in untreated mice, and in Treg-depleted and non-depleted tumor-bearing mice. Both the tumor-draining and non-draining LN were examined. Averages+SEM from 3 independent experiments each with 5 mice per group are shown.

Since Treg are present at relatively high frequencies in both tumor tissue and LN (Figure 1B), we hypothesized that one of their targets for suppression would be resident DC. TIDC were identified as CD45^+^ and CD11c^hi^, and comprised CD11b^hi^ and CD11b^int^ subpopulations (Figure 2A). Both subpopulations expressed CD40, CD86 and MHCII, although expression was highest on CD11b^hi^ DC. In addition to the CD11c^hi^ subpopulations, tumors also contained a population of cells expressing markers consistent with plasmacytoid DC [3]; these cells represented only a small proportion of TIDC and were not further examined.

As shown in Figure 1F, B16.OVA tumors grew more slowly in Treg-depleted mice. To obtain tumors of similar size, Treg-depleted and non-depleted mice were injected with tumor 1–2 days apart. When TIDC were compared between these two groups, no difference could be observed in DC frequency, number per milligram of tumor tissue, or phenotype (Figures 2B, 2C, 2D). Results shown in Figures 2B, 2C, and 2D refer to CD11b^hi^ cells, but similar results were obtained with the CD11b^int^ population (data not shown). CD40, CD86 and MHCII were similarly expressed in the TIDC from both treatment groups, both as percentage of positive cells (Figure 2D) and as Mean Fluorescence Intensity (MFI) in the total population (data not shown). The relative frequencies of CD11b^hi^ and CD11b^int^ TIDC subpopulations were also similar in untreated and Treg-depleted mice (data not shown). Treg depletion did not affect the number or maturation status of DC in the LN, nor was there a difference in maturation status between DC in the draining and non-draining LN, both when measured as percent positive cells or as MFI (Figure 2E and data not shown). Thus, Treg depletion does not appear to affect the numbers or phenotype of DC in the tumor, or DC phenotype in the tumor-draining LN.

### Treg depletion does not improve the ability of TIDC to induce proliferation of tumor-specific T cells *in vitro*

**Figure 3:**
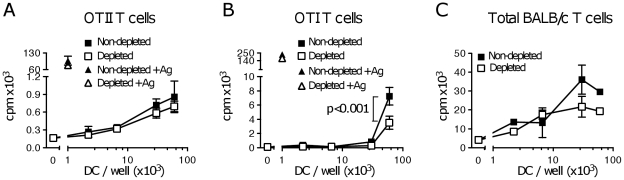
Treg depletion does not increase the ability of TIDC to stimulate T cell proliferation *ex vivo*. C57BL/6 mice were treated with PC61 or left untreated, and injected with B16.OVA s.c. After 14–17 days CD45^+^ CD11c^hi^ TIDC were sorted and titrated in triplicate into cultures containing (A) OTII T cells, (B) OTI T cells or (C) BALB/c allogeneic T cells. As a positive control, specific peptide antigen was loaded on 10^3^ TIDC/well before co-culture with T cells where indicated. Proliferation was measured 3 days later. Each panel shows one of 3 independent experiments that gave similar results. Average ± SEM are shown. *p* was calculated using a two-way ANOVA test with a Bonferroni post-test.

CD45^+^CD11c^+^ cells, including both CD11b^hi^ and CD11b^int^ DC subpopulations, were electronically sorted from tumor cell suspensions to a high purity (>96%) and used to stimulate the proliferation of OVA-specific CD4^+^ OTII and CD8^+^ OTI T cells *in vitro*. To test the presentation of tumor antigen taken up by DC within the tumor context, no further antigen was added to the assay except in the positive controls. As previously reported [3], in the absence of added antigen, sorted DC were unable to stimulate proliferation of OVA-specific OTII cells (Figure 3A) and induced only minimal proliferation of OVA-specific OTI cells (Figure 3B). The low proliferation of OTI cells was not antigen-specific, as it was observed even when DC were prepared from B16.F1 tumors that do not express OVA [3]. Sorted DC were otherwise able to present synthetic peptide to OTI and OTII cells [3], or added OVA protein to OTII cells (data not shown), to induce proliferation *in vitro*. When TIDC were prepared from the tumors of Treg-depleted mice, no increase in the proliferation of OTI or OTII cells was observed (Figure 3A and 3B).

Although TIDC were unable to present OVA taken up within the tumor context, they appeared functional as they could stimulate the proliferation of specific T cells in the presence of peptide (Figure 3A and 3B), and proliferation of allogeneic BALB/c T cells *in vitro* (Figure 3C). In both cases, Treg depletion did not improve the response.

### Treg depletion does not improve the proliferation of tumor-specific OTI T cells in the tumor-draining LN

Since removing DC from the tumor environment might reverse the effects of Treg on DC *in situ*, we also examined the presentation of tumor-derived antigen using an *in vivo* assay. Presentation of tumor antigen in the draining LN is thought to reflect migration of DC from the tumor site, which is known to be defective [1] but might improve if the frequency of Treg in the tumor is decreased. Presentation in the LN may also reflect transfer of antigen from migratory to resident DC [41], which would bypass antigen presentation defects in tumor-derived DC.

**Figure 4:**
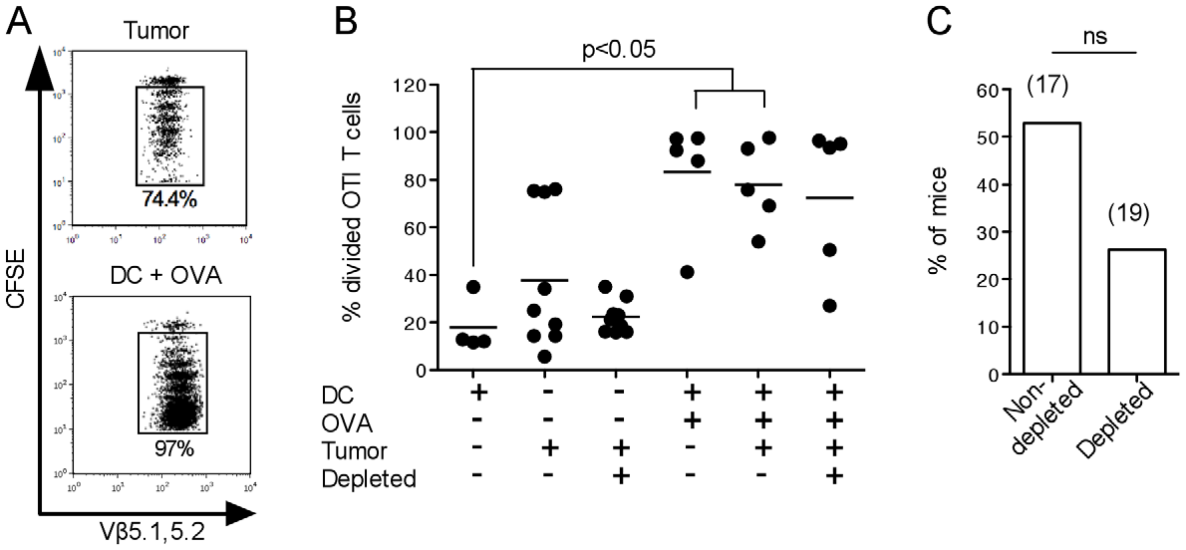
Treg depletion does not affect the proliferation of tumor-specific T cells *in vivo*. C57BL/6 mice were treated with PC61 or left untreated, and injected with B16.OVA s.c. After 13–16 days each mouse was injected with 1.5×10^6^ naïve, CFSE-labeled OTI T cells. LN were removed 3 days later and OTI T cell proliferation was determined by flow cytometry. (**A**) Representative dot plots of proliferating OTI T cells in the draining LN of tumor-bearing mice (top panel) or non tumor-bearing mice immunized with OVA_257–264_ -loaded DC (bottom panel). The percent divided cells is shown. (**B**) Division of OTI T cells in LN draining the tumor or DC immunization site. Where both tumor and DC were given, the LN draining the immunization site was examined. Horizontal lines show the average percentages of divided cells. The graph is representative of 4 independent experiments with 5–10 mice per group per experiment. (**C**) Percentage of mice showing OTI T cell proliferation in the tumor-draining LN. The total number of mice (n) in each group is shown. Data are compiled from 3 separate experiments; ns, not significant by a Fisher's exact probability test.

Naïve, CFSE-labeled OTI T cells were transferred into Treg-depleted or non-depleted tumor-bearing mice, and their proliferation in tumor-draining LN was compared 3 days later. A representative dot plot of OTI proliferation in tumor-bearing mice is shown in the top panel of Figure 4A. As a control, we used non tumor-bearing mice immunized with DC+OVA_257–264_; a representative dot plot of OTI proliferation in the LN draining the DC injection site is shown in the lower panel of Figure 4A. Little OTI T cell proliferation was observed in non tumor-bearing mice that received DC not loaded with OVA_257–264_ (Figure 4B). A high level of proliferation was detected in some of the tumor-bearing mice, which was antigen-specific [3], but always lower in extent than the proliferation observed in the positive controls (Figure 4A). This proliferation was not increased in magnitude (Figure 4B) or frequency (Figure 4C) by Treg depletion. Surprisingly, the proportion of mice where T cells proliferated was even decreased in Treg-depleted mice, however, this decrease was not statistically significant (p = 0.17 by a Fishers exact probability test) and further experiments are required to establish the reproducibility of this observation. High proliferation was observed in all mice injected with OVA_257–264_-loaded DC, indicating that the transferred T cells were functional and were not adversely affected by the presence of tumor, or by PC61 treatment (Figure 4B).

Similar experiments were carried out to examine the proliferation of CD4^+^ OTII cells. As previously reported using both low and highly immunogenic models, no CD4^+^ OTII T cell proliferation was detected in tumor-bearing mice [3], [10]. Additional experiments in PC61-treated mice showed that proliferation was not restored by Treg depletion (data not shown).

### The proliferation of OTI T cells is not increased in the tumor-draining LN of RAG1^−/−^ compared to C57BL/6 mice

To determine whether the residual Treg population seen in PC61-treated mice (Figures 1A and 1B) might be sufficient to affect DC function in tumor-bearing mice, we examined tumor-specific T cell proliferation in RAG1^−/−^ mice, which lack all T cells including Treg. TIDC were present in tumors from RAG1^−/−^ mice at frequencies similar to those in C57BL/6 tumors (Figure S2A) and expressed similar, although slightly less mature, activation phenotypes (Figure S2B). TIDC phenotype in RAG1^−/−^ mice was not significantly affected by the transfer of purified CD8^+^ T cells or CD4^+^CD25^+^ Treg one day before tumor inoculation (data not shown).

**Figure 5:**
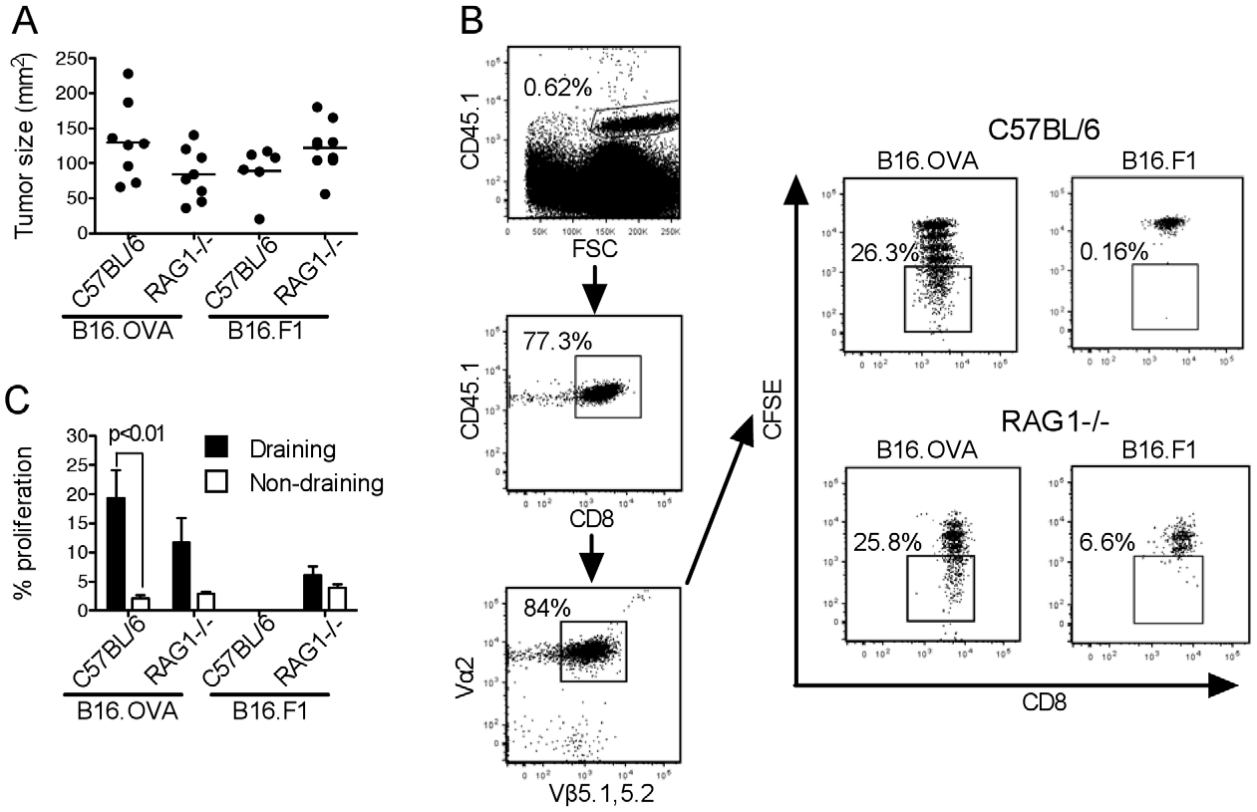
The proliferation of tumor-specific OTI T cells in tumor-bearing C57BL/6 and RAG1^−/−^ hosts is similar. C57BL/6 and RAG1^−/−^ mice were injected with B16.OVA or B16.F1 s.c.. After 15 days each mouse was injected with 1.5×10^6^ naïve, CD8-enriched, CFSE-labeled OTI T cells and 1.5×10^6^ naïve, CD25-depleted, CD4-enriched, CFSE-labeled OTII T cells. LN were removed 3 days later and T cell proliferation was determined by flow cytometry. (**A**) Tumor size at the experimental endpoint (day 18); each dot corresponds to one mouse and the horizontal line shows the average tumor size. (**B**) Gating strategy used to identify the donor OTI T cell population in the draining LN. Antigen-specific division was determined on the basis of CFSE dilution as shown in the right-hand dot plots. (**C**) Antigen-specific OTI T cell division in the tumor-draining and non-draining LN of C57BL/6 and RAG1^−/−^ mice. Bars show the average percentage of cells that divided more than 3 times, +SEM. Data is from one experiment with 6–9 mice per group. *p* was calculated using two-way ANOVA with a Bonferroni post-test.

To examine the effect of Treg on tumor-specific T cell proliferation *in vivo*, C57BL/6 and RAG1^−/−^ mice were inoculated with either B16.OVA or OVA-negative B16.F1 tumors. Both tumors grew at a similar rate in the two strains (Figure 5A) suggesting that in C57BL/6 hosts there was little spontaneous T cell response to the B16 tumors. Tumor-bearing mice received CFSE-labeled OTI and CD25-depleted OTII T cells and OVA-specific proliferation was compared in the tumor-draining (Figure 5B) and non-draining LN 3 days later. As shown in Figure S3, the OTII populations contained low frequencies of Foxp3^+^ T cells that were further decreased by CD25 depletion; about half of the remaining cells expressed the transgenic TCR and were presumably OVA-specific.

It has been shown that in lymphopenic hosts OTI T cells undergo 2–3 rounds of homeostatic proliferation in the absence of antigen [42] therefore, to exclude homeostatic proliferation, only OTI cells that had divided more than 3 times were considered. This division was mostly antigen-specific, as it occurred in the LN draining B16.OVA tumors but not in those draining B16.F1. Specific OTI T cell proliferation was not increased in RAG1^−/−^ mice compared to C57BL/6 (Figure 5C), suggesting that Treg were not inhibiting OTI T cell division in C57BL/6 mice. Similarly, little or no division of OTII T cells was observed in C57BL/6 or RAG1^−/−^ mice injected with either B16.OVA or B16.F1 tumors (data not shown), again suggesting that lack of division was not due to the presence of Treg in C57BL/6 mice.

## Discussion

The recruitment of Treg to tumors is an early event in tumor establishment [43]. The negative correlation observed between the presence of Treg in tumors and survival outcome [44] suggests that these Treg are probably assisting in the evasion of the immune response. As also reported in other tumor models [17], [40] we found that even small B16.OVA melanoma tumors contained higher frequencies of CD25^+^ Foxp3^+^ Treg than blood, and that tumor-infiltrating Treg had suppressive activity *in vitro*. Somewhat surprisingly, we also found that depletion of Treg appeared to have no detectable effect on the frequency, phenotype or function of TIDC. A partial but functionally relevant depletion via injection of the anti-CD25 monoclonal antibody PC61, or a more profound depletion by the use of RAG1^−/−^ hosts, both failed to affect TIDC phenotype. Similarly, defective proliferation of tumor-specific CD4^+^ and CD8^+^ T cells in tumor-draining LN, which may reflect presentation of tumor antigen by DC migrating from the tumor to the LN, was not improved in these mice. Therefore, CD4^+^CD25^+^ Treg appeared to have no effect on the presentation of tumor antigen by DC in this tumor model, possibly suggesting that other regulatory populations that are not affected by PC61 treatment were involved.

It has been shown that Treg suppress the responses of many cell types, using a variety of mechanisms including direct killing [21], [24], [35]. Using the B16.OVA melanoma model, we found no significant difference between the numbers, frequency or relative proportions of TIDC populations in Treg-depleted and non-depleted mice. The frequency and absolute number of DC in the LN were also similar (data not shown). These observations fail to support the possibility that in this tumor model CD4^+^CD25^+^ Treg inhibit DC function by directly or indirectly affecting their survival.

Experiments using both *in vitro* and *in vivo* models have shown that, in the presence of Treg, DC express decreased levels of the maturation markers CD40, CD80, CD86 and MHCII (reviewed in [21]). The DC subpopulation/s that are involved in presentation of tumor antigen in B16.OVA melanoma tumors have not been precisely identified, however, in other tumor models multiple DC subpopulations can present tumor antigen in the draining LN [45]. On this basis, we chose to include in our analysis all CD11c^hi^ DC subpopulations in these tissues. While DC in B16.OVA tumors and LN appeared relatively mature, there was no effect of Treg depletion on the proportion of TIDC subpopulations, or on any of the DC activation markers tested. Both TIDC and Treg localized mainly to the peripheral area of B16 tumors and could often be found in close proximity to each other (data not shown), suggesting that the two populations had the potential to interact within the tumor context. Additional experiments showed that Treg depletion also did not rescue or enhance the ability of TIDC to stimulate the proliferation of tumor-specific T cells *in vitro*, or the ability of DC in tumor-draining LN to stimulate T cell proliferation *in vivo*. Taken together, our results suggest that Treg had little or no effect on TIDC in this tumor model.

Our inability to demonstrate an effect of PC61 treatment on DC function could not be explained by incomplete Treg depletion, as the same results were observed using a RAG1^−/−^ model in which Treg depletion was profound. Transfer of CD8^+^ OTI and CD25-depleted CD4^+^ OTII T cells into RAG1^−/−^ tumor-bearing mice 3 days before analysis provided a model in which significant conversion of CD4^+^ T cells into Treg was unlikely to occur, but CD4^+^ T cells could provide help to DC thereby rescuing potential defects in DC function due to lymphopenic conditions [46]. Even in this situation, the proliferation of OTI or OTII cells was not improved, suggesting the presentation of tumor antigen in the draining LN was similar in RAG1^−/−^ hosts and C57BL/6 mice.

It is conceivable that the effects of Treg on DC might have been missed in our experiments, if these only occur early during tumor development, at time points where the limited amount of tumor tissue available makes the study of TIDC more difficult. We believe that this is unlikely to be the case, as we and others have shown that as the tumor increases in size, the frequency of Treg increases and the microenvironment becomes more suppressive. Under these increasingly hostile conditions, it is doubtful that suppression could be spontaneously overcome, and any effects that the Treg may have had on the DC at early timepoints are likely to still be evident at later stages of tumor growth.

A recent paper used an OVA-expressing 3-methylcholanthrene (MCA)-induced tumor to report that tumor antigen-specific Treg could directly kill DC in the tumor draining LN [35]. A detailed comparison of those findings with ours is not possible, as the presence and localization of Treg and DC within tumors, and the ability of tumor-derived DC to present OVA to T cells, were not characterized [35]. The observation that Treg rapidly kill DC presenting tumor antigen in the LN might suggest that DC in MCA tumors do not come into contact with Treg, and can therefore survive unaffected until they reach the LN. A differential ability of TIDC from MCA or B16 tumors to present OVA antigen in the context of MHCII, and induce activation of CD4^+^ T cells and Treg, might also contribute to the different findings in these studies.

It has been reported that antigen derived from normal tissues can be presented to self-reactive CD4^+^ T cells in the draining LN and elicit a response [47], [48]. In tumors this is not always the case, and reports by other Authors and ourselves [3], [49]–[51] suggest that TIDC may be unable to induce productive activation of CD4^+^ T cells. A reduced ability of TIDC to activate conventional CD4^+^ T cells might also imply a reduced ability of these DC to activate Treg, as suggested by experiments showing that in inflamed tissue the same DC drive cytokine production by both CD4^+^ Teff and Treg [52]. If this is the case, the results reported in this study might be explained by hypothesizing that Treg accumulate in the tumor, but fail to recognize the appropriate ligands in the context of DC to become activated to effector function. As it has been shown by us (Figure 1F) and by other Authors [53] that Treg depletion delays the growth of B16 tumors (Figure 1F), and improves the anti-tumor efficacy of both prophylactic [38] and therapeutic [54] DC immunotherapy, Treg must be suppressing anti-tumor immune responses by mechanisms other than acting on DC. For example, reports using intravital microscopy of mouse LN have demonstrated that Treg can directly act on T cells to suppress immune responses [55]. In our studies, Treg depletion did not improve CD4^+^ or CD8^+^ T cell proliferation, implying that any direct effect of Treg on T cells might have an impact on the quality of the T cell response, rather than the quantity. The defective DC function we and others observe in tumor-bearing mice [3], [6], [7], [9]–[12] may then be attributable to other suppressive factors present in the hostile tumor environment, such as other regulatory T cell populations, myeloid-derived suppressor cells, or anti-inflammatory cytokines, which may act directly or indirectly on DC in tumor and/or draining LN. Indeed, a recent study by Herber et al [13] showed that DC in tumor bearing hosts upregulate the scavenger receptor A resulting in increased lipid uptake, and impaired antigen presenting function.

In conclusion, we show that CD4^+^CD25^+^ Treg appear to have little effect on the number, phenotype and function of TIDC in B16 melanoma, suggesting that the delayed tumor growth observed in Treg-depleted mice is unlikely to be due to improved DC function. The question of whether or not Treg affect DC in a tumor model is complex and highly relevant to designing improved therapeutic anti-tumor vaccines. This study suggests that, in order to be optimally effective, regulatory T cell depletion should be used in association with other forms of immunotherapy

## Supporting Information

Figure S1 **Identification of Treg in the blood of untreated and PC61 treated mice.** Foxp3GFP mice were treated with PC61 or left untreated, and injected with B16.OVA tumors s.c. (**A**) The frequencies of Treg in blood from non-depleted mice, or mice depleted of Treg by PC61 treatment, were determined by flow cytometry. The lower panels show the Foxp3GFP^+^ populations as identified in the top panels. Each panel refers to an individual representative mouse. (**B**) Mice were treated with PC61 or left untreated, and tail bled over time to monitor the frequency of Tregs in blood by flow cytometry. CD25^+^Foxp3^+^ cells were gated as shown in the lower part of panel A. Average ± SEM for groups of 5 mice are shown.
(EPS)

Figure S2 **TIDC frequency and phenotype are similar in C57BL/6 and RAG1^−/−^ mice.** C57BL/6 and RAG1^−/−^ mice were injected with B16.OVA tumors s.c. Tumors were removed 17 days later and analyzed by flow cytometry. (**A**) Frequencies of CD11c^hi^ TIDC in tumors were compared between C57BL/6 and RAG1^−/−^ mice. *p* was calculated using the Student's one tailed t test. (**B**) Frequencies of TIDC expressing the indicated maturation markers were compared between C57BL/6 and RAG1^−/−^ mice. Values of *p* were calculated using a two-way ANOVA test with a Bonferroni post-test. Data is from one experiment with 9–10 mice per group.
(EPS)

Figure S3 **Frequencies of Foxp3^+^ T cells in naïve OTI and OTII cell populations before transfer into RAG1^−/−^ hosts.** (**A**) Frequencies of CD8^+^ T cells (top panels) and Foxp3^+^ Treg (lower panels) in the total OTI lymphocyte population were determined before and after enrichment for CD8^+^ cells. (**B**) Frequencies of CD4^+^ T cells (top panels) and Foxp3^+^ Treg (lower panels) in the total OTII lymphocyte population were determined before and after the cells were depleted of CD25^+^ cells and enriched for CD4^+^ cells. The frequency of OVA-specific (Valpha2^+^, Vbeta5.1,5.2^+^) Foxp3^+^ Treg in the CD4-enriched population is shown in the right lower panel. Foxp3 expression was determined by intracellular staining.
(EPS)
